# Digital Versus in‐Person Pre‐Anaesthetic Assessment and Postoperative Outcomes in Low‐Risk Patients: An Observational Study

**DOI:** 10.1111/aas.70393

**Published:** 2026-11-13

**Authors:** Mathilde Nellemann, Sofie Amalie Bosholdt, Moritz Kilian German Denneborg, Camilla Meno Kristensen, Constance Thyra Giertz Christiansen, Janus Christian Jakobsen, Rasmus Hesselfeldt, Morten Hylander Møller, Andreas Møgelmose, Markus Harboe Olsen, Katrine Bredahl Buggeskov

**Affiliations:** ^1^ Department of Anaesthesia, Surgery and Trauma Centre Copenhagen University Hospital—Rigshospitalet Copenhagen Denmark; ^2^ Copenhagen Trial Unit, Centre for Clinical Intervention Research, the Capital Region Copenhagen University Hospital, Rigshospitalet Copenhagen Denmark; ^3^ Department of Regional Health Research, the Faculty of Health Sciences University of Southern Denmark Odense Denmark; ^4^ Department of Intensive Care Copenhagen University Hospital—Rigshospitalet Copenhagen Denmark; ^5^ Department of Clinica Medicine University of Copenhagen Copenhagen Denmark; ^6^ Visual Analysis and Perception Lab Aalborg University Aalborg Denmark; ^7^ Department of Neuroanaesthesiology, the Neuroscience Centre Copenhagen University Hospital—Rigshospitalet Copenhagen Denmark

## Abstract

**Background:**

Pre‐anaesthetic assessment is essential for identifying perioperative risk and optimising patient safety. Digital alternatives, including video consultation and structured electronic health record (EHR) review, may improve efficiency, but comparative evidence regarding postoperative safety remains limited. This study aimed to evaluate postoperative outcomes across pre‐anaesthetic assessment through EHR review, video consultation, and in‐person assessment. We hypothesised that patients selected for digital pre‐anaesthetic assessment within a structured triage system would have similar low 30‐day rates of major postoperative complications compared with in‐person assessment.

**Methods:**

We conducted a single‐centre cohort study at Copenhagen University Hospital—Rigshospitalet, Denmark, including adult patients undergoing elective otorhinolaryngologic, oral, or maxillofacial surgery. Patients underwent pre‐anaesthetic assessment by one of three modalities: in‐person consultation, EHR review, or video consultation. The primary outcome was a composite of major postoperative complications within 30 days, including all‐cause mortality, intensive care unit admission, bleeding requiring transfusion, infection requiring antibiotics, or hospital readmission. A multivariable generalised linear model with a log‐link function was used to estimate adjusted relative risks (RRs) with 95% confidence intervals (CIs).

**Results:**

A total of 862 patients were included (in‐person *n* = 300; EHR review *n* = 300; video consultation *n* = 262). Patients in the in‐person group were older and had a higher comorbidity burden. Using in‐person as reference, neither video consultation nor EHR review showed a significantly different risk of major postoperative complications. Hospital readmission was lower in both digital groups in pairwise comparisons with the in‐person group. Results were consistent across sensitivity analyses, including inverse probability weighting and win ratio analysis.

**Conclusion:**

Patients selected for digital pre‐anaesthetic assessment had low rates of major postoperative complications. However, substantial baseline differences between groups indicate that assessment modality was strongly linked to clinical triage and baseline patient risk, thereby limiting direct comparison between pathways. Thus, these findings should not be interpreted as evidence of equivalence or non‐inferiority between pathways.

**Editorial Comment:**

Preoperative anesthesia assessment is managed in different ways, based on local preferences logistics and resource availability, and perceived perioperative risk for complications. This study assessed this process for planned surgery in section at a university hospital. Those during a number of recent years chosen for in‐person preoperative assessment (for any reason) were compared to those who had some form of digital assessment chosen by the anesthesia personnel. The selection process for in‐person preoperative assessment seemed to identify those who had a higher chance of a major postoperative complication.

## 1 Background

Pre‐anaesthetic assessment is a cornerstone of perioperative care, aimed at identifying and optimising patient‐related risk factors to minimise postoperative morbidity and mortality [1]. Prior to surgery, patients undergo this structured evaluation of perioperative risks and potential challenges, including difficult airway management [2]. Such assessments help reduce surgical cancellations and delays, minimise peri‐ and postoperative complications, and alleviate patient anxiety, thereby improving overall patient satisfaction [3]. In 2024, more than 357,000 general anaesthetic procedures were performed in Danish hospitals, representing a 22% increase compared with 2022 [4, 5]. Globally, more than 300 million patients undergo anaesthesia annually [6]. Traditionally, pre‐anaesthetic assessments are conducted as an in‐person consultation lasting 5–20 min, corresponding to up to 400,000 clinician hours annually in Denmark. In the context of an ageing population and increasing healthcare demands, the sustainability of this model is uncertain [7].

Digital transformation offers opportunities to redesign this workflow and improve efficiency. Alternative approaches, such as video consultations and structured reviews of electronic health records, may streamline pre‐anaesthetic assessments while maintaining the quality of care. These approaches may reduce hospital visits and time spent travelling to and from the hospital. For clinicians, they may optimise specialist resource allocation by reserving in‐person evaluations for higher‐risk cases. From a system perspective, such approaches may improve efficiency, reduce costs, and promote equitable access to standardised preoperative assessments regardless of geographical location.

Despite these potential benefits, patient safety remains paramount. Perioperative complications continue to be a significant public health concern, with reported mortality rates of approximately 1% [8] and complication rates around 12% [9]. Patients who experience complications may require prolonged hospitalisation, develop functional limitations, and have reduced long‐term survival [10]. Thorough pre‐anaesthetic assessment is therefore essential for identifying modifiable risk factors and optimising perioperative management.

Current European Society of Anaesthesiology and Intensive Care (ESAIC) guidelines emphasise structured preoperative evaluation but do not clearly define the role of digital or remote assessment modalities [11]. While video‐based assessments have been studied [12, 13], evidence comparing multiple assessment modalities, including electronic health record review, remains limited. Observational studies within real‐world settings can complement randomised trials by evaluating implementation and safety in routine clinical practice [14, 15].

This study aimed to evaluate postoperative outcomes across the three pre‐anaesthetic assessment pathways as implemented in routine clinical practice. We hypothesised that patients selected for digital pre‐anaesthetic assessment within a structured triage system would have low 30‐day rates of major postoperative complications, comparable to those observed among patients in person.

## 2 Methods

This study was conducted as a single‐centre cohort study based on routinely collected data from Copenhagen University Hospital—Rigshospitalet, Copenhagen, Denmark. The study examined postoperative outcomes across pre‐anaesthetic assessment pathways, including in‐person consultation, video consultation, and EHR review. A detailed protocol was published prior to analysis [16].

The observation period spanned 2019–2025, reflecting the sequential implementation of pre‐anaesthetic assessment modalities: in‐person consultations (2019–2020), electronic health record (EHR) review (2021–2022), and video consultation (2024–2025). To the authors' knowledge, no major structural changes in perioperative pathways were formally introduced during this period, although minor and undocumented changes in workflow cannot be excluded.

### 2.1 Ethics and Approvals

The project was initiated as a quality assurance evaluation of the implementation of video‐based and electronic health record‐based pre‐anaesthetic assessments, to assess whether these modalities were associated with acceptable perioperative safety and workflow outcomes compared with in‐person assessment. The study did not involve intervention or deviation from standard care and was therefore classified as a quality assurance study, requiring approval only from the management of Copenhagen University Hospital—Rigshospitalet. According to Danish regulations, the project did not require approval from the research ethics committee or informed consent. Approval to access patient information for the purpose of this quality assurance study was granted by the management of Rigshospitalet under reference number: P‐2024‐15419, on 24‐09‐2024.

The study adhered to the principles of the Declaration of Helsinki and institutional data protection standards, and data were handled in accordance with the General Data Protection Regulation (GDPR) and the Danish Data Protection Act.

### 2.2 Settings

The cohort included adult patients (≥ 18 years) undergoing elective otorhinolaryngologic, oral or maxillofacial surgery at Copenhagen University Hospital—Rigshospitalet. To ensure the safety of the initial implementation of the digital assessment modalities, these were initially used in patients considered to be at relatively low risk of postoperative complications. Digital assessment modalities, such as video consultation and EHR review, were not randomly assigned but determined through routine clinical triage based on pre‐screening and clinician judgement. Patients were eligible for digital assessment (electronic health record review or video consultation) if pre‐screening via a standardised health questionnaire indicated low perioperative risk (see Table S4). Eligibility for video consultation additionally required sufficient functional capacity to participate in remote assessment, including adequate hearing and vision, as well as the ability to use digital devices and attend scheduled appointments. All included patients had to be American Society of Anesthesiologists (ASA) physical status classification I–II to reduce variability in comorbidities among patients in the different groups. Patients not eligible for the study included those undergoing acute surgery or patients already included in the study. To ensure independent observations, only the first eligible surgical episode per patient was included.

### 2.3 Data Sources

Data were derived from:

- In‐person cohort: a random sample of all patients with an ASA physical status of I–II undergoing elective surgery in the otorhinolaryngologic, oral or maxillofacial surgery departments at Copenhagen University Hospital—Rigshospitalet in the years 2019–2020
- Video cohort: data derived from a quality assurance dataset of video consultations established by one of the co‐authors with patients undergoing otorhinolaryngologic, oral or maxillofacial surgery at Copenhagen University Hospital—Rigshospitalet in 2024–2025
- Electronic health record review cohort: data derived from a quality assurance dataset established by one of the co‐authors with patients undergoing otorhinolaryngologic, oral or maxillofacial surgery at Copenhagen University Hospital—Rigshospitalet in 2021–2022.

Patient baseline characteristics, records from the pre‐anaesthetic assessment and postoperative outcomes within 30 days after surgery were manually extracted for each patient through the electronic medical record system “Sundhedsplatformen” (Epic Systems, Verona, Wisconsin, USA).

A full list of baseline variables is provided in Table S1.

### 2.4 Outcomes

#### 2.4.1 Primary Outcome

A 30‐day composite of major postoperative complications across a spectrum of severity.

- All‐cause mortality
- Any admission to the intensive care unit
- Bleeding requiring transfusion
- Infection requiring antibiotics
- Hospital readmission for any cause

#### 2.4.2 Secondary Outcomes

- Change in anaesthetic plan on the day of surgery.
- Change in airway management from post‐induction tracheal intubation to awake/sedated airway management procedure.
- Change from non‐invasive to invasive blood pressure monitoring.
- Initiation of continuous vasopressor infusion at induction.
- Number of tracheal intubation attempts.
- Same‐day surgery cancellation.
- Delay in surgical start time (minutes).
- Days hospitalised within 30 days after surgery, excluding the day of surgery.

Each component of the primary outcome was also analysed separately as an exploratory outcome. Definitions of the primary and secondary outcomes are provided in Tables S2 and S3.

### 2.5 Bias and Confounding

Potential sources of bias include confounding by indication due to triage‐based selection of assessment modality, selection bias related to eligibility for digital assessment, and temporal confounding due to sequential implementation of modalities.

### 2.6 Sample Size Estimation

Based on a baseline complication rate of approximately 11.8% [9], a sample size of 300 patients per group would provide approximately 80% power to detect a relative difference of 54% or more. Each cohort included 300 patients, except the video consultation cohort, in which 262 patients met the inclusion criteria.

### 2.7 Statistical Analysis

A full description of the statistical analysis can be found in the prepublished protocol [16]. Data management and statistical analyses were performed using the latest stable version of R version 4.5.0 (R Core Team, Vienna, Austria) and data integrity was ensured by performing validation checks for duplicates, implausible values, and outliers. The primary comparison was between in‐person and video consultation, whereas comparisons involving EHR review were exploratory.

#### 2.7.1 Primary Analyses

The primary outcome was a dichotomous composite endpoint of major postoperative complications occurring within 30 days, defined as all‐cause mortality, any admission to the intensive care unit, bleeding requiring transfusion, infection requiring antibiotics, and hospital readmission. There were no missing data for the primary outcome; therefore, multiple imputation was not performed [17, 18].

Secondary outcomes were same‐day surgery cancellation, changes in anaesthesia plan, changes in airway management, changes from non‐invasive to invasive blood pressure monitoring, and initiation of continuous vasopressor infusion at induction. All were analysed as dichotomous variables using multivariable generalised linear models with log‐link function, and with assessment modality as the primary exposure. Effect estimates were reported as relative risk (RRs) with corresponding 95% confidence intervals.

The primary models were adjusted for three prespecified covariates selected for their clinical relevance and potential to confound the association between assessment modality and postoperative outcomes [3, 19]: specialty (otorhinolaryngologic or oral and maxillofacial procedures), age, and comorbidity burden, measured manually using the Charlson Comorbidity Index (CCI) [20]. Additional variables, including sex, ASA classification, and smoking status, were examined in subgroup analyses to explore potential influences on outcome measures.

Since assumptions for linear regression models for continuous outcomes were not met, results were presented using the Wilcoxon rank‐sum test with median difference and Hodges‐Lehmann confidence intervals.

Definitions of the primary and secondary outcomes are provided in Tables S2 and S3.

#### 2.7.2 Subgroup Analyses

Subgroup analyses were performed for the primary outcome across the following prespecified subgroups:

- Age group ≥ 65 versus > 65 years.
- Surgical specialty: otorhinolaryngologic versus oral/maxillofacial procedures.
- Comorbidity burden: Charlson Comorbidity Index 0–1 versus ≥ 2.
- Sex: male versus female.
- Body mass index (BMI): below versus above median BMI.
- Smoker status: current smoker versus non‐current smoker.
- ASA classification: I versus II.

For each subgroup, an interaction term between assessment modality and the subgroup variable was included in the univariable generalised linear model using log‐link function for the primary outcome. The subgroup analyses were hypothesis‐generating and should be interpreted cautiously, particularly given the limited statistical power. Because there was no overlap in surgery years between groups, no subgroup analysis was performed for calendar year.

#### 2.7.3 Sensitivity Analysis

Win ratio analysis [21] was performed as a sensitivity analysis using a prespecified hierarchical ordering of the components of the primary outcome. In addition, an unadjusted sensitivity analysis was performed. To further investigate whether systematic differences between the groups exist, inverse probability weighting (IPW) was also performed as a sensitivity analysis.

## 3 Results

A total of 862 patients were included in the study, of whom 300 underwent in‐person pre‐anaesthetic assessment, 262 underwent video consultation, and 300 underwent EHR review (Figure 1). Patients in the in‐person group were older and had a higher comorbidity burden (Table 1) compared with the video group and the EHR group. The proportion of patients in the ASA II class was substantially higher in the in‐person group (74.3%) than in the video group (39.7%) and the EHR group (34.7%) (Table 1). Baseline characteristics differed substantially between groups, reflecting the triage‐based allocation of patients to assessment pathways.

**FIGURE 1:**
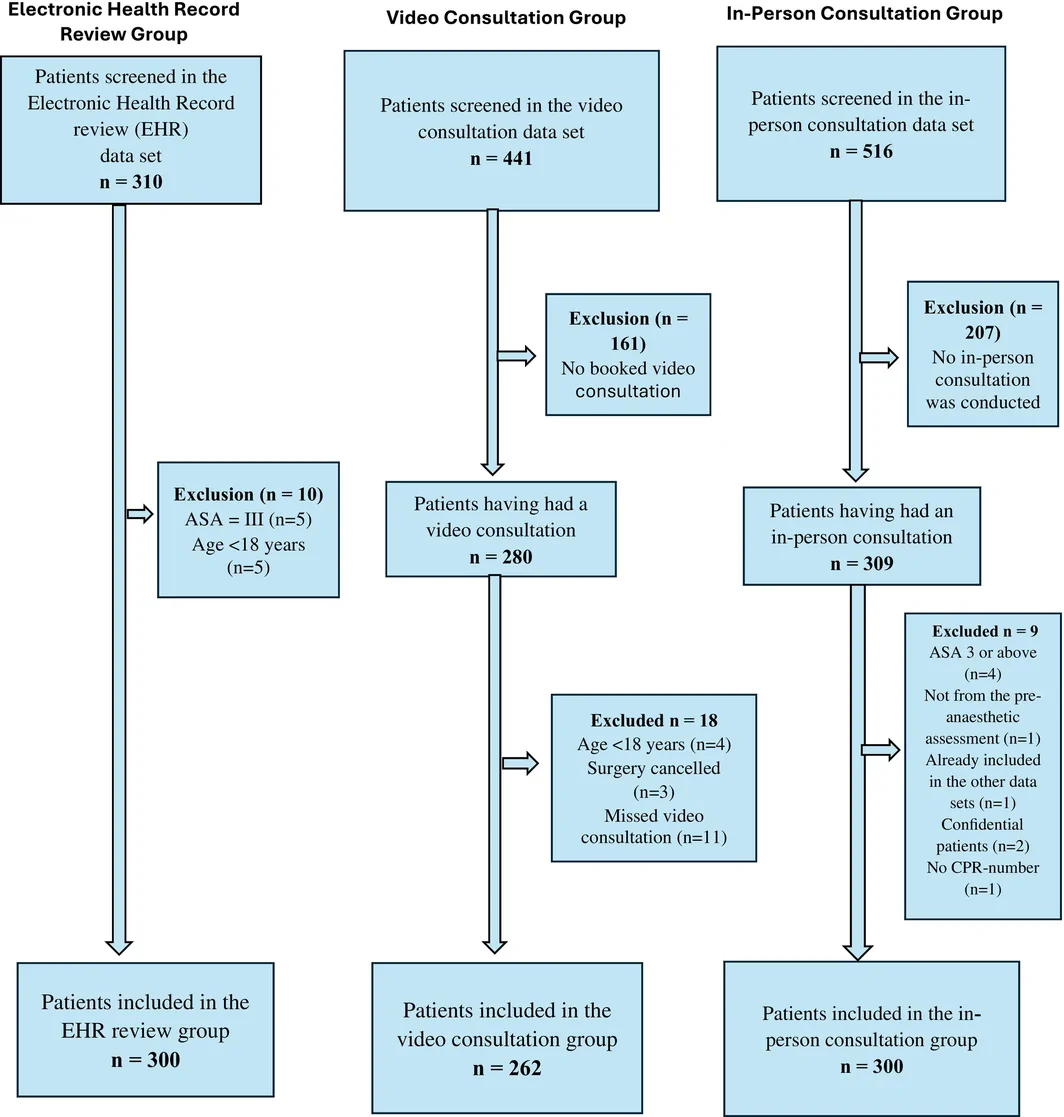
Flow diagram of patient inclusion.

**TABLE 1:** Baseline characteristics for the three pre‐anaesthetic assessment modalities: In‐person consultation, electronic health record (EHR) review, and video consultation.

	In‐person consultation *n* = 300 (%)	EHR review *n* = 300 (%)	Video consultation *n* = 262 (%)
Age—median [IQR]	49.5 [30.0–63.0]	29.5 [23.0–46.0]	31.5 [25.0–41.7]
Sex—Male	146 (48.7)	149 (49.7)	144 (55.0)
BMI—median [IQR]	24.8 [22.10–27.75]	23.5 [21.30–26.20]	24.0 [22.00–26.89]
Missing	117 (39)	6 (2)	4 (1.5)
ASA classification—II	223 (74.3)	104 (34.7)	104 (39.7)
CCI—median [IQR]	1 [0.00–2.00]	0 [0.00–1.00]	0 [0.00–0.00]
Missing	3 (1)	11 (3.7)	1 (0.4)
Smoking status			
Current	44 (14.7)	66 (22.0)	51 (19.5)
Former	57 (19.0)	61 (20.3)	52 (19.8)
Never	74 (24.7)	152 (50.7)	128 (48.9)
Missing	125 (41.7)	21 (7.0)	31 (11.8)
Mallampati			
Missing	2 (0.7)	70 (23.3)	6 (2.3)
I	175 (58.3)	138 (46.0)	158 (60.3)
II	86 (28.7)	75 (25.0)	83 (31.7)
III	28 (9.3)	13 (4.3)	13 (5.0)
IV	9 (3.0)	4 (1.3)	2 (0.8)
Mouth opening			
Missing	5 (1.7)	72 (24.0)	8 (3.1)
< 2.5 cm	1 (0.3)	0 (0.0)	0 (0.0)
< 4 cm	14 (4.7)	12 (4.0)	4 (1.5)
> 4 cm	280 (93.3)	216 (72.0)	250 (95.4)
Jaw thrust			
Missing	7 (2.3)	82 (27.3)	8 (3.1)
Limited	20 (6.7)	18 (6.0)	2 (0.8)
Normal	273 (91.0)	200 (66.7)	252 (96.2)
Thyromental distance			
Missing	115 (38.3)	124 (41.3)	87 (33.2)
< 6.5 cm	6 (2.0)	3 (1.0)	0 (0.0)
> 3 FB	95 (31.7)	49 (16.3)	96 (36.6)
> 6.5 cm	84 (28.0)	124 (41.3)	79 (30.2)
Neck movement			
Missing	7 (2.3)	77 (25.7)	9 (3.4)
Limited	16 (5.3)	2 (0.7)	1 (0.4)
Full	277 (92.3)	221 (73.7)	252 (96.2)
Detailed specialty			
Oral/maxillofacial—possibly malignant	0 (0.0)	2 (0.7)	0 (0.0)
Oral/maxillofacial—non‐malignant	86 (28.7)	213 (71.0)	43 (16.4)
Ear‐nose‐throat surgery—possibly malignant	129 (43.0)	1 (0.3)	2 (0.8)
Ear‐nose‐throat surgery—non‐malignant	85 (28.3)	84 (28.0)	217 (82.8)
Year of surgery			
2019	93 (31.0)	0 (0.0)	0 (0.0)
2020	207 (69.0)	0 (0.0)	0 (0.0)
2021	0 (0.0)	195 (65.0)	0 (0.0)
2022	0 (0.0)	105 (35.0)	0 (0.0)
2024	0 (0.0)	0 (0.0)	260 (99.2)
2025	0 (0.0)	0 (0.0)	2 (0.8)

Abbreviations: ASA = American Society of Anaesthesiologists (ASA) physical status classification, BMI = Body mass index, CCI = Charlson Comorbidity Index.

Despite these baseline differences, postoperative complication rates remained low across all pathways. Compared with in‐person assessment, the relative risk of major postoperative complications was 0.70 (95% CI 0.44–1.10; *p* = 0.125) for video consultation and 0.88 (95% CI 0.57–1.35; *p* = 0.561) for EHR review (Table 2). No deaths were observed in any group. ICU admission and bleeding requiring transfusion were rare events across all modalities.

**TABLE 2:** Primary analysis of the composite of major postoperative complications.

Characteristic	RR	95% CI	*p*
Group			
In‐person	—	—	
EHR‐review	0.88	0.57–1.35	0.561
Video	0.70	0.44–1.10	0.125
CCI	1.01	0.82–1.25	0.920
Age	1.00	0.98–1.02	0.915
Surgical specialty			
ENT	—	—	
OMFS	1.00	0.67–1.49	0.993

Abbreviations: CCI = Charlson Comorbidity Index, CI = Confidence Interval, ENT = Ear‐nose‐throat, OMF = Oral/maxillofacial, RR = Relative Risk.

In analyses of individual components of the primary outcome, hospital readmission within 30 days was more frequent in the in‐person group (11.0%) compared with both the video (4.2%) and EHR groups (3.3%). This corresponded to a lower risk of readmission for video consultation (RR 0.33, 95% CI 0.16–0.68; *p* = 0.003) and EHR review (RR 0.40, 95% CI 0.18–0.84; *p* = 0.002) compared with in‐person assessment (Table 3). Patients assessed via video consultation had fewer days hospitalised compared with in‐person assessment (median difference 1 day, 95% CI 0–1; *p* < 0.001), whereas no significant difference was observed between in‐person and EHR review.

**TABLE 3:** Pairwise comparisons of primary, secondary, and exploratory outcomes between assessment modalities.

In‐person consultation vs. video consultation				
	In‐person *n* = 300 (%)	*n* = 262 (%)	Generalised linear model RR (95% CI)	*p*
Major postoperative complications	50 (16.7)	30 (11.5)	0.66 (0.41–1.06)	0.084
All‐cause mortality	0 (0.0)	0 (0.0)	—	—
ICU‐admission	1 (0.3)	2 (0.8)	9.19 (0.58–335.68)	0.144
Bleeding	0 (0.0)	0 (0.0)	—	—
Infection	28 (9.3)	21 (8.0)	0.82 (0.45–1.50)	0.528
Readmission	33 (11.0)	11 (4.2)	0.33 (0.16–0.68)	0.003
Change in anaesthetic plan	67 (22.3)	59 (22.5)	1.37 (0.91–2.05)	0.128
Change in airway management	0 (0.0)	0 (0.0)	—	—
Change in blood pressure monitoring	2 (0.7)	0 (0.0)	—	—
Initiation of continuous vasopressor infusion	0 (0.0)	0 (0.0)	—	—
Same‐day surgery cancellation	7 (2.3)	4 (1.5)	0.40 (0.10–1.43)	0.166
Delay in surgical start time	0 (0.0)	0 (0.0)	—	—

	In‐person *n* = 300	*n* = 262	Wilcoxon rank‐sum test median diff. (95% HLCI)	*p*
Number of tracheal intubation attempts—median [IQR]	1.00 [1.00;1.00]	1 [1.00;1.00]	0.00 (0.00;0.00)	0.277
missing	6 (2%)	1 (0.4%)		
Hospital‐free days—median [IQR]	29.00 [29.00–30.00]	30.00 [30.00–30.00]	1.00 (0.00–1.00)	0.000

In‐person consultation vs. EHR review				
	In‐person *n* = 300 (%)	EHR‐review *n* = 300 (%)	Generalised linear model RR (95% CI)	*p*
Major postoperative complications	50 (16.7)	43 (14.3)	0.97 (0.63–1.51)	0.905
All‐cause mortality	0 (0.0)	0 (0.0)	—	—
ICU‐admission	1 (0.3)	0 (0.0)	—	—
Bleeding	0 (0.0)	0 (0.0)	—	—
Infection	28 (9.3)	36 (12.0)	1.23 (0.72–2.15)	0.446
Readmission	33 (11.0)	10 (3.3)	0.40 (0.18–0.84)	0.020
Change in anaesthetic plan	67 (22.3)	73 (24.3)	0.98 (0.66–1.45)	0.905
Change in airway management	0 (0.0)	0 (0.0)	—	—
Change in blood pressure monitoring	2 (0.7)	1 (0.3)	0.82 (0.05–12.81)	0.888
Initiation of continuous vasopressor infusion	0 (0.0)	0 (0.0)	—	—
Same‐day surgery cancellation	7 (2.3)	5 (1.7)	0.42 (0.13–1.38)	0.154
Delay in surgical start time	0 (0.0)	0 (0.0)	—	—

	In‐person *n* = 300	EHR‐review *n* = 300	Wilcoxon rank‐sum test median diff. (95% HLCI)	*p*
Number of tracheal intubation attempts—median [IQR]	1.00 [1.00;1.00]	1 [1.00;1.00]	0.00 (0.00–0.00)	0.709
Missing	6 (2%)	11 (3.7%)		
Hospital‐free days—median [IQR]	29.00 [29.00;30.00]	30 [28.00;30.00]	0.00 (0.00–0.00)	0.072
Missing	0 (0%)	(0.3%)		

EHR review vs. video consultation				
	EHR‐review *n* = 300 (%)	*n* = 262 (%)	Generalised linear model RR (95% CI)	*p*
Major postoperative complications	43 (14.3)	30 (11.5)	0.97 (0.56–1.65)	0.904
All‐cause mortality	0 (0.0)	0 (0.0)	—	—
ICU‐admission	0 (0.0)	2 (0.8)	—	—
Bleeding	0 (0.0)	0 (0.0)	—	—
Infection	36 (12.0)	21 (8.0)	0.84 (0.44–1.58)	0.593
Readmission	10 (3.3)	11 (4.2)	0.98 (0.36–2.77)	0.969
Change in anaesthetic plan	73 (24.3)	59 (22.5)	1.71 (1.13–2.58)	0.011
Change in airway management	0 (0.0)	0 (0.0)	—	—
Change in blood pressure monitoring	1 (0.3)	0 (0.0)	—	—
Initiation of continuous vasopressor infusion	0 (0.0)	0 (0.0)	—	—
Same‐day surgery cancellation	5 (1.7)	4 (1.5)	0.53 (0.12–2.52)	0.411
Delay in surgical start time	0 (0.0)	0 (0.0)	—	—

	EHR‐review *n* = 300	*n* = 262	Wilcoxon rank‐sum test median diff. (95% HLCI)	*p*
Number of tracheal intubationattempts—median [IQR]	1 [1.00;1.00]	1 [1.00;1.00]	0.00 (0.00–0.00)	0.150
Missing	11 (3.7%)	1 (0.4%)		
Hospital‐free days—median [IQR]	30 [28.00;30.00]	30.00 [30.00;30.00]	—	—
Missing	1 (0.3%)	0 (0%)		

Subgroup analyses did not demonstrate statistically significant interactions between assessment modality and prespecified subgroups, including age, sex, BMI, ASA classification, or comorbidity burden. Estimates were generally consistent across subgroups, although precision was limited due to small numbers in several strata (Figure 2).

**FIGURE 2:**
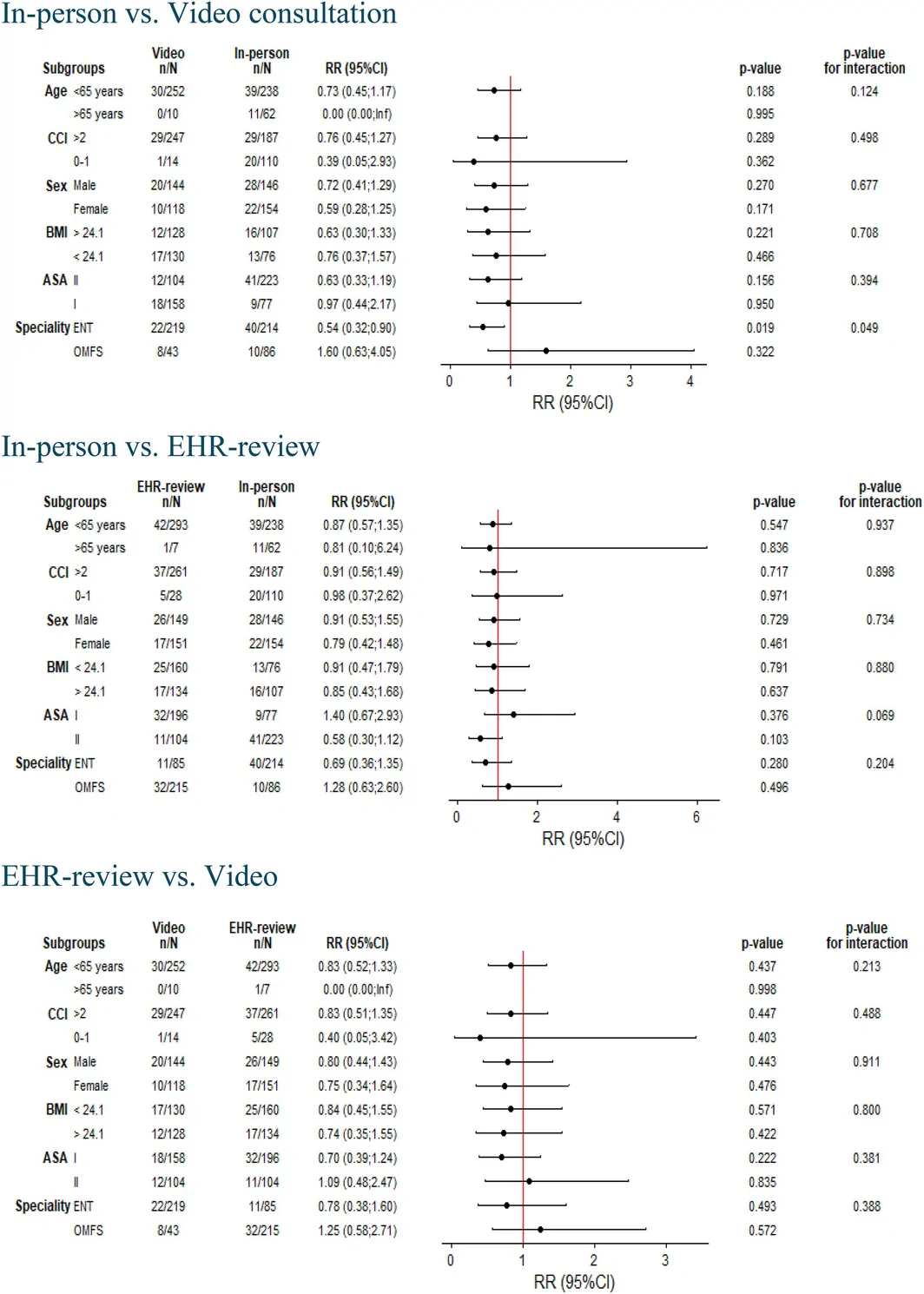
Pairwise subgroup analysis for the primary composite outcome of major postoperative complications for in‐person versus video consultation, in‐person versus EHR review, and EHR review versus video consultation. ASA = American Society of Anaesthesiologists (ASA) physical status classification, BMI = Body mass index, CCI = Charlson Comorbidity Index, ENT = Ear‐nose‐throat surgical specialty, OMF = Oral/maxillofacial surgery specialty.

Win ratio analyses showed no statistically significant differences between modalities. For the comparison between in‐person assessment and video consultation, the win ratio was 1.49 (95% CI 0.94–2.37; *p* = 0.093). Corresponding win ratios were 1.17 (95% CI 0.77–1.78; *p* = 0.47) for in‐person versus EHR review and 1.28 (95% CI 0.79–2.07; *p* = 0.32) for EHR review versus video consultation (Figure S1). Finally, inverse probability weighting yielded similar estimates as the primary multivariable analysis (Figure S2).

## 4 Discussion

This study evaluated postoperative outcomes across triaged pre‐anaesthetic assessment pathways in routine clinical practice. Among 862 patients undergoing elective otorhinolaryngologic, oral, or maxillofacial surgery, major postoperative complications were uncommon across all pre‐anaesthetic assessment pathways. However, patients in the in‐person consultation cohort were older and had a higher comorbidity burden than those assessed digitally, reflecting the triage‐based implementation of the assessment modalities. Thus, the findings primarily describe outcomes among patients selected for each pathway rather than providing a direct comparison of the safety of the assessment modalities.

Hospital readmission occurred less frequently among the digital groups. This finding should be interpreted cautiously, as it is likely driven by confounding by indication and selection bias rather than a true effect of assessment modality. Adjustment for surgical specialty did not meaningfully affect the results, suggesting that differences in case mix between otorhinolaryngologic and oral/maxillofacial procedures were unlikely to explain the findings.

The low incidence of complications among patients selected for video consultation or EHR review is reassuring and supports the feasibility of incorporating digital assessment into a structured triage system for selected low‐risk patients. Importantly, however, this study does not establish that digital assessment itself is as safe as in‐person assessment. Rather, the findings support the feasibility of selecting appropriately screened, lower‐risk patients for digital assessment while retaining in‐person assessment for patients who require more comprehensive evaluation.

Randomised controlled trials are needed to determine whether different assessment modalities have comparable clinical outcomes and to evaluate patient satisfaction, resource use, and cost‐effectiveness. Ongoing work within our research group aims to contribute to this evidence base through complementary systematic reviews [22] and randomised investigations [23] such as the VIDFACE trial.

### 4.1 Strengths and Limitations

This study has several strengths. First, the study used routinely collected clinical data from several surgical departments within Copenhagen University Hospital—Rigshospitalet, providing a realistic depiction of current practice and real‐world implementation within a European healthcare system. Moreover, the inclusion of patients undergoing otorhinolaryngologic, oral or maxillofacial surgery—specialties in which airway management is often particularly relevant [24]—may increase the relevance of the findings to other surgical populations with similar airway‐related challenges. By including three models of pre‐anaesthetic assessment, the study shed light on the postoperative outcomes across a spectrum of existing clinical practices. The use of 30‐day clinically meaningful outcomes and multiple analytical approaches, including multivariable adjustment, inverse probability weighting, and win ratio analysis, further strengthened the robustness of the findings. In addition, this study also contributes to the limited literature on structured EHR review as a pre‐anaesthetic assessment modality.

Despite these strengths, several limitations should be acknowledged. The principal limitation is confounding by indication and selection bias. Digital assessment was implemented through clinical triage, and patients eligible for digital assessment were pre‐screened as lower perioperative risk. The marked differences in age, ASA classification, and comorbidity burden indicate that the groups represented different clinical risk strata created by triage rather than directly comparable populations. Although patients in the in‐person consultation group were required to be ASA I or II and statistical adjustment and propensity‐based methods were applied, these methods cannot fully account for residual confounding, unmeasured clinical judgement, or the structural separation between groups. Direct causal comparisons between modalities are therefore limited. In addition, eligibility for video consultation required sufficient functional and digital capacity to participate in remote assessment. This may introduce further selection bias, as patients in this group may differ in cognitive function, sensory abilities, and digital literacy.

A further limitation is the temporal confounding, as the assessment modalities were implemented sequentially. Although no major structural changes to the perioperative pathway were identified, smaller changes in workflow, staffing, or practice over the study period cannot be ruled out. Because the assessment modality and calendar period were closely linked, the ability to distinguish temporal effects from assessment modality effects was limited.

The single‐centre design also limits generalisability, and findings may be most applicable to healthcare systems with similar triage‐based pre‐anaesthetic assessment pathways. The study was conducted exclusively at Copenhagen University Hospital—Rigshospitalet, and the implementation of digital pre‐anaesthetic assessment was influenced by the local workflows, organisational structure, and experience and acceptance of the healthcare professionals within the department. Furthermore, Copenhagen University Hospital—Rigshospitalet is a highly specialised university hospital, and its patient population, surgical case mix, and available resources may differ from those of smaller or more rural hospitals. More broadly, the Danish healthcare system also has a high degree of digitalisation. The acceptance and feasibility of digital pre‐anaesthetic assessment may therefore differ in other countries or healthcare systems with different organisational structures, patient populations, and levels of digitalisation.

Other limitations include unmeasured factors, such as clinician judgement, which may have influenced both modality selection and outcome, and the relatively modest sample size, which limited the ability to detect smaller clinically relevant differences. In addition, minor complications may have been underreported in routine documentation. Moreover, missingness was substantial for some baseline variables, particularly BMI and smoking status in the in‐person group, which limited the interpretation and statistical power of the subgroup analyses.

Finally, the study was not designed as a non‐inferiority analysis, and the observational design limits causal inference. Although multivariable adjustment and propensity‐based methods can address measured differences between groups, they cannot fully account for unmeasured confounding or replace randomisation. Randomised controlled trials, such as the VIDFACE trial [23], are therefore needed to determine whether digital and in‐person assessment provide comparable clinical outcomes.

Digital assessment also has practical limitations that are relevant to implementation. For patients, video consultation requires adequate digital literacy, access to suitable equipment, and sufficient hearing and vision. Some patients may also prefer face‐to‐face interaction, particularly when discussing concerns related to anaesthesia and surgery. For clinicians, the absence of physical examination may also limit assessment of certain clinical features, including frailty and general physical appearance, while technical difficulties, late attendance, or failure to attend scheduled appointments may affect workflow efficiency.

EHR review has different limitations. It does not involve direct interaction between the patient and the anaesthesiologist, and relevant information may be incomplete or dependent on information documented by other healthcare professionals. Patients may therefore have questions or concerns that are not identified during the review. These practical considerations highlight that digital assessment should not necessarily be viewed as a universal replacement for in‐person assessment, but rather as an additional approach within an appropriately structured clinical pathway.

## 5 Conclusion

In this real‐world cohort, patients selected for digital pre‐anaesthetic assessment had low rates of major postoperative complications. However, substantial baseline differences between assessment groups reflect clinical triage and limited direct comparison between the different pre‐anaesthetic modalities. These findings support the potential feasibility of digital pre‐anaesthetic assessment within structured, risk‐stratified pathways, but do not establish equivalence, non‐inferiority, or causal safety advantage compared with in‐person assessment.

## Author Contributions

All authors will meet the recommendations for authorship defined by The International Committee of Medical Journal Editors.

## Funding

The authors have nothing to report.

## Conflicts of Interest

Katrine Breddahl Buggeskov has received funding from BETA.HEALTH, Innovation Fund Denmark, and Holger & Ruth Hesses mindefond (Holger & Ruth Hesse's Memorial Fund) outside the submitted work.

## Supporting information


**Table S1:** Baseline variables and definitions.
**Table S2:** Definition of primary outcome, a composite of major postoperative complications including.
**Table S3:** Definition of secondary outcome.
**Table S4:** Pre‐screening health questionnaire.
**Figure S1:** Sensitivity analysis using win ratio.
**Figure S2:** Sensitivity analysis using inverse probability weighting.

## Data Availability

Data can be made available upon reasonable request and only after relevant approvals.
