# Supplementary material for: Digital Versus in‐Person Pre‐Anaesthetic Assessment and Postoperative Outcomes in Low‐Risk Patients: An Observational Study
Source: Acta Anaesthesiol Scand. 2026 Nov 13;71(1):e70393. doi: 10.1111/aas.70393 (PMC13760547; doi:10.1111/aas.70393)
Supplement: Supplementary file 1 [file AAS-71-0-s001.docx]

**Supplementary appendix**

**Table S1**: Baseline variables and definitions

| Baseline Variables | Definition |
| --- | --- |
|  |  |
| Age | Years from birth to the date of surgery |
| Sex | Biological sex (male/female) |
| BMI | Weight in kg divided by height in meters squared |
| Type of surgery | Otorhinolaryngologic vs oral and maxillofacial procedures |
| Surgery duration | Described in minutes or hours |
| Comorbidities | Neurological, respiratory, cardiovascular, gastrointestinal, endocrine or musculoskeletal disease |
| Smoking | Daily, in the past or never |
| Mallampati | Number 1-4 |
| Mouth opening | >2.5 cm, < 4 cm or > 4 cm |
| Neck mobility | Full or limited |
| Thyromental distance | > 6.5 cm or < 6.5 cm |
| Ability to protrude jaw | Full or limited |
| ASA classification | Number I-II |
| Previous problems with anaesthesia | PONV, allergic reactions, cardiovascular complications and more |

**Table S2:** Definition of primary outcome, a composite of major postoperative complications including:

| Primary outcome | Definition |
| --- | --- |
|  |  |
| 30-day postoperative mortality | Yes/ No |
| 30-day post-surgery admission to intensive care | Yes/ No |
| 30-day postoperative bleeding requiring blood transfusion | Yes/ No |
| 30-day post-surgery infection requiring antibiotics | Yes/ No |
| 30-day post-surgery hospital readmission | Yes/ No |

**Table S3**: Definition of secondary outcome

| Secondary outcomes | Definition |
| --- | --- |
|  |  |
| Same-day surgery cancellations | Yes/ No |
| Changes in the anaesthesia plan | Yes/ No |
| Changes in airway management | Yes/ No |
| Changes from non-invasive to invasive blood pressure monitoring | Yes/ No |
| Initiation of continuous vasopressor infusion at induction | Yes/ No |
| Delay in surgical start time | Minutes |
| Intubation attempts | Number 1-5 |
| Days hospitalised within 30 days of surgery | Number of days within 30 days after surgery, excluding the day of surgery |

**Table S4**

**Pre-screening health questionnaire**

**Table S4: Health questionnaire**

Health questionnaire used in the structured screening to assess whether patients were eligible for digital assessment pathways such as video consultation or electronic health record (EHR) review

| **If answered no to all questions, the patient is eligible for a digital pre-anaesthetic assessment. If answered yes to one or more questions, the patient will have an in-person consultation with an anaesthesiologist** | **Yes** | **No** |
| --- | --- | --- |
| 1. **Do you wish to speak to an anaesthesiologist?** |  |  |
| 2. **Do you have trouble opening your mouth?** |  |  |
| 3. **Have you had problems with general anaesthesia before?** |  |  |
| 4. **Do you have a congenital heart disease?** |  |  |
| 5. **Are you under 15 or above the age of 75?** |  |  |
| 6. **Do you need to hold a break when having to go up the stairs to second floor?** |  |  |
| 7. **Do you have sleep apnoea?** |  |  |
| 8. **Do you have medically treated diabetes?** |  |  |
| 9. **Do you take either Wegovy or Ozempic?** |  |  |
| **10. Do you take 5 or more medications?** |  |  |
| **11. Do you suffer from severe pain requiring morphine or other opioids?** |  |  |
| 12. **Do you use cannabis or other recreational drugs?** |  |  |
| 13. **Do you take any other blood thinner than acetylsalicylic acid?** |  |  |
| 14. **Are you pregnant?** |  |  |
| 15. **Have you had any surgery or radiation therapy to the mouth or neck region?** |  |  |
| 16. **Do you have a hiatal hernia or had stomach surgery?** |  |  |
| 17. **Are you not comfortable enough in Danish and English and will require an interpreter?** |  |  |
| **Objective examination** | | |
| 1. **Systolic blood pressure > 180 mmHg** |  |  |
| 2. **Diastolic blood pressure > 110 mmHg** |  |  |
| 3. **BMI > 35** |  |  |

**Figure S1**: Sensitivity analysis using win ratio

**Figure S2**: Sensitivity analysis using inverse probability weighting

| Estimate | OR | CI 2.5 | CI 97.5 |
| --- | --- | --- | --- |
| Video vs. In-person (MV) | 0.684 | 0.374 | 1.258 |
| EHR-review vs. In-person (MV) | 0.891 | 0.508 | 1.582 |
| Video vs. In-person (IPW) | 0.664 | 0.378 | 1.169 |
| EHR-review vs. In-person (IPW) | 0.952 | 0.565 | 1.603 |
